# Luciferase-based reporting of suicide gene activity in murine mesenchymal stem cells

**DOI:** 10.1371/journal.pone.0220013

**Published:** 2019-07-18

**Authors:** Dario Gerace, Rosetta Martiniello-Wilks, Rosaline Habib, Ann Margaret Simpson

**Affiliations:** 1 The School of Life Sciences and the Centre for Health Technologies, University of Technology Sydney, Sydney, Australia; 2 Translational Cancer Research Group, University of Technology Sydney, Sydney, Australia; Università degli Studi della Campania, ITALY

## Abstract

Due to their ease of isolation, gene modification and tumor-homing properties, mesenchymal stem cells (MSCs) are an attractive cellular vehicle for the delivery of toxic suicide genes to a variety of cancers in pre-clinical models. In addition, the incorporation of suicide genes in stem cell-derived cell replacement therapies improves their safety profile by permitting graft destruction in the event of unexpected tumorigeneses or unwanted differentiation. Due to the functional requirement of ATP for the *Firefly luciferase* gene *Luc2* to produce light, luciferase-based reporting of cytotoxicity can be engineered into potential cell therapies. Consequently, we nucleofected mammalian expression plasmids containing both the *Luc2* and the yeast fusion cytosine deaminase uracil phosphoribosyltransferase (*CDUPRT*) genes for expression in murine MSCs to assess luciferase as a reporter of suicide gene cytotoxicity, and MSC as vehicles of suicide gene therapy. *In vitro* bioluminescence imaging (BLI) showed that following the addition of the non-toxic prodrug fluorocytosine (5-FC), *CDUPRT*-expressing MSCs displayed enhanced cytotoxicity in comparison to *Luc2* reporter MSC controls. This study demonstrates the utility of luciferase as a reporter of *CDUPRT-*mediated cytotoxicity in murine MSC using BLI.

## Introduction

MSCs were originally identified by Friedenstein *et al*, in 1976 as a fibroblast-like cell population [1]. The definition of MSCs has evolved over time due to the changing understanding of MSC biology. Currently, the International Society for Cellular Therapies (ISCT) has defined MSCs as a heterogeneous stem cell population characterised by; (i) adherence to plastic under standard culture conditions; (ii) a fibroblast-like morphology; (iii) the capacity to differentiate into osteocytes, chondrocytes and adipocytes; (iv) lack of expression of haematopoietic markers CD11b, CD14, CD34, CD19 or CD79a, CD45, HLA-DR and the vascular marker CD31 [2–6]; (v) expression of CD13, CD44, CD54, CD73, CD90, CD105, CD146, CD166, CD200, SCA-1 and STRO-1 [2, 4–7]. Due to their ease of isolation and genetic modification, *ex vivo* expanded MSCs have been assessed in the pre-clinical and clinical setting as vehicles for therapeutic gene delivery.

Suicide gene therapy is grounded on the concept of delivering a bacterial or viral gene to mammalian cells, whose enzyme product is able to convert a non-toxic prodrug to its toxic form resulting in cell death [8]. Consequently, this controllable system of cell death has been assessed as an alternative therapy to traditional cancer treatments such as chemotherapy and radiation therapy. Suicide gene therapy has been assessed in the treatment of leukaemia [9], prostate cancer [10] and breast cancer [11] amongst many others. A number of systems exist that function via enzymatic conversion of a prodrug to its lethal form. The most commonly assessed systems are the herpes simplex virus thymidine kinase gene [12, 13] with ganciclovir as the pro-drug, and the *Escherichia coli* cytosine deaminase gene (*CD*) [14, 15] with 5-FC as the prodrug. Following conversion of the non-toxic prodrug 5-FC to its lethal form 5-fluorouracil (5-FU), apoptosis is induced in targeted cells via interference of the mitochondrial pathway [14]. The *CD* system has been further improved by fusion with the uracil phosphoribosyltransferase (UPRT) gene (*CDUPRT*) which facilitates the conversion of the toxic 5-FU to 5-FU monophosphate, further sensitizing 5-FU-resistant tumor cells to low concentrations of 5-FU [16].

Bioluminescence imaging (BLI) is a novel method of assessing cellular cytotoxicity by exploiting the fact that dying cells stop producing bioluminescence, as luciferase activity is ATP-dependent. As a result, BLI has been demonstrated to be superior to the traditional Chromium-51 release cytotoxicity assay, due to its increased signal-to-noise ratio and faster kinetics [17]. In this study, we developed an *in vitro* luciferase reporter system for monitoring the cytotoxicity of the *CDUPRT* gene, engineered to be expressed in murine MSCs. We confirmed the cytotoxic function of *CDUPRT* in engineered MSCs and identified the minimum concentration at which 5-FC becomes detrimental to health of normal MSCs. The results from this study demonstrate the utility of *in vitro* luciferase reporting of *CDUPRT*-mediated MSC cytotoxicity and confirms the potential for MSC-derived suicide therapy.

## Materials and methods

### Sourcing of animals

NOD mice were sourced from the Animal Resources Centre (WA, Australia). All animal work was approved by the UTS Animal Care and Ethics Committee (ACEC 2011-447A; ACEC 2009-244A), and complied with the Australian code for the care and use of animals for scientific purposes [18].

### MSC isolation and cell culture

Three MSC isolations were performed using a procedure adapted from published protocols [19–22]. Briefly, bone marrow was flushed from the femurs of twenty female NOD mice (6–8 weeks old), and the cell pellet was resuspended in standard medium (α-MEM, 1% (v/v) 100x Penicillin/Streptomycin/L-Glutamine (P/S/G) with 20% (v/v) Fetal Bovine Serum (FBS)) (all sourced from Gibco, Thermofisher), and incubated at 37°C/5% CO_2_. Plastic-adherent stromal cells were sub-cultured for two passages (with epiphyses) prior to FACS.

Passage 2 plastic-adherent stromal cells (5x10^5^ cells) were resuspended in sorting buffer (1x HBSS, 5% (v/v) FBS) and stained with 0.2mg/ml rat anti-mouse CD45 monoclonal antibody (mAb) conjugated to allophycocyanin (APC) (BD Pharmingen, USA) and 0.2mg/ml rat anti-mouse Ly6 (Sca-1) mAb conjugated to phycoerythrin (PE) (BD Pharmingen, USA). Stained stromal cells sorted via FACS at the Advanced Cytometry Facility (Centenary Institute, Sydney, Australia) on a BD FACSAria II flow cytometer and analysed using BD FACSDiva software (Version 6.1.3). The stromal cells were sorted into CD45^-^/Ly6^+^ (MSCs) and CD45^+^/Ly6^+^ (double positive) cell populations. Sorted cells were resuspended in standard MSC medium and incubated at 37°C/5% CO_2_. Following cell attachment, 10ng/ml basic fibroblast growth factor (bFGF) was added to the medium.

### MSC viability and clonogenicity

For cell viability, MSCs and double positive cells at early (P3-15) passage number were seeded in 24-well plates (2.5x10^3^ cells/well) (Falcon BD Biosciences, San Jose, USA) in triplicate, and maintained in standard MSC medium for 15 days, with medium replenished weekly. Cell viability was assessed by Trypan Blue (0.4% v/v; Gibco, Thermofisher) exclusion. Total cell and viable cell numbers were determined and represented as mean ± standard deviation (SD) for each time point (n = 3).

For clonogenicity assays, MSCs and double positive cells at early (P3-15) passage number were seeded in 10cm^2^ tissue culture treated plates (5x10^2^ cells/plate) (Falcon BD Biosciences), and maintained in standard MSC medium for 10 days. Colonies were stained with 0.4% v/v methylene blue in methanol and counted by microscopy. Data were represented as mean colony count per 5000 cells ± SD (n = 3). Standard MSC medium was replenished weekly.

### Differentiation assays

#### Adipogenesis

Early (P3-15) passage cells were seeded in standard MSC medium in 24-well plates (2.5x10^4^ cells/well) in triplicate and grown to 80–90% confluence. The medium was subsequently replenished with either adipogenic control or differentiation medium as previously described [23]. The cells were stained with 0.2% (w/v) Oil Red O in methanol (Fronine, Sydney, Australia) and semi-quantitatively scored as previously described [23]. Values were expressed as counts per cm^2^ and were represented as mean ± SD (n = 3).

#### Osteogenesis

Early (P3-15) passage cells were seeded in standard MSC medium in 24-well plates (1.25x10^4^ cells/well) in triplicate and grown to 90–95% confluence. The medium was subsequently replenished with either osteogenic control or differentiation medium as previously described [23]. The cells were stained with 2% (w/v) Alizarin Red S (pH 4.1) (Fronine) and semi-quantitatively scored as previously described [23]. Values were expressed as counts per cm^2^ and were represented as mean ± SD (n = 3).

#### Chondrogenesis

Early (P3-15) passage cells were seeded in standard MSC medium in 24-well plates (1.25x10^4^ cells/well) and grown to 90% confluence. The medium was subsequently replenished with either control (MesenCult-ACF Chondrogenic Differentiation Basal Medium (STEMCELL Technologies, Vancouver, Canada), 2mM L-glutamine) or differentiation (MesenCult-ACF Chondrogenic Differentiation Basal Medium, 2mM L-glutamine, MesenCult-ACF 20X Chondrogenic Differentiation Supplement) medium and incubated at 37°C/5% CO_2_ for 18 days. On day 18, the cells were fixed in 10% neutral buffered formalin and stained with Alcian blue solution (8x, pH2.5) (Sigma-Aldrich, Sydney, Australia). Chondrogenesis was visualised by Alcian blue staining of filamentous glycosaminoglycans.

### Construction of mammalian expression plasmids

The manipulation of genetic material and the generation of genetically modified organisms was approved by the UTS Biosafety Committee (2001-19-R-GC; 2009-02-R-GC). Briefly, the luciferase reporter gene *Luc2* (*Photinus pyralis*), encoded within the vector pGL4.20 (*Luc2*/Puro) (Promega, USA), was digested with the restriction enzymes EcoRV-HF and BamHI-HF (New England Biolabs, USA), and ligated into the mammalian dual expression plasmid pVITRO2-hygro-mcs (InvivoGen, USA), to generate the plasmid pVITRO2-*Luc2*. The *CDUPRT* gene encoded by the plasmid pORF5-Fcy::Fur (InvivoGen, USA), was digested with EcoRI-HF and NheI-HF (New England Biolabs, USA), and ligated into the pVITRO2-*Luc2* plasmid, to generate the plasmid pVITRO2-*Luc2*/*CDUPRT*.

### Nucleofection

Early passage MSCs (1x10^6^ cells/reaction) were nucleofected with 5μg of either pVITRO2-*Luc2* or pVITRO2-*Luc2*/*CDUPRT*, and 2μg pmax-GFP (Lonza, USA) using the Nucleofector II device (Lonza, USA) according to the manufacturer’s instructions. Following nucleofection, the cells were returned to culture in standard MSC medium at 37°C/5% CO_2_ for one week. Stable clones were then selected with 200μg/ml Hygromycin B (Thermofisher Scientific) over a two-week period.

### Morphological analysis

Images of four fields of view at 10x or 20x magnification were acquired at early passage number using a Leica DM microscope (Leica Microsystems, Weltzar, Germany), and processed using the image processing software, Leica Application Suite (V4.4.0) (Leica Microsystems). Scale bars on figures are equivalent to 100μm.

### *In vitro* luciferase assay

A linear concentration of MSC-*Luc2*, MSC-*Luc2*/*CDUPRT* and MSC-*Luc2/LacZ ID7* (positive control) (1x10^4^-6x10^5^ cells/well) were seeded in 96-well ViewPlate microplates in triplicate (n = 3). The cells were incubated at 37°C/5% CO_2_ overnight, and imaged on the IVIS Lumina II (PerkinElmer, USA) the following day following the addition of 150μg/ml D-Luciferin (Gold Biotechnology, USA). BLI was performed at multiple time-points (t = 0, 15, 30, 60, 90, 120 and 180 min) to determine the stability of luciferase activity over a 3-hour period. For quantification, a region of interest (ROI) was manually selected using the Living Image (Version 3.1) software. BLI intensity values are represented as the mean average radiance ± SDs (p/s/cm^2^/sr). The following *in vitro* BLI acquisition settings were used: Incubation time; 2 min, Exposure time; 30 sec, F stop; 1, Field of view; D, Binning; Small.

### 5-FC and 5-FU *in vitro* cytotoxicity assay

Early passage MSC-*Luc2* and MSC-*Luc2*/*CDUPRT* (5x10^2^ cells/well) were transferred to half of a 96-well ViewPlate microplate (PerkinElmer, USA) (n = 12 total) respectively and incubated at 37°C/5% CO_2_ for 24 hours. The following day, a 2-fold serial dilution of 0-2mg/ml 5-FC (Invivogen, USA) and a 10-fold serial dilution of 0–0.1mg/ml 5-FU (Invivogen, USA) were prepared in standard MSC medium, and added to the 96-well ViewPlate microplates (+/-5-FC; n = 3 and +/-5-FU; n = 3). The plates were subsequently incubated at 37°C/5% CO_2_ for 5 days, after which the plates were imaged for luciferase expression on the IVIS Lumina II using the in *vitro* BLI acquisition settings. BLI intensity values are represented as the mean average radiance ± SDs (p/s/cm^2^/sr).

### Statistical analysis

All statistical analysis was performed using GraphPad Prism 7 software. Values are represented as means ± SDs or SEMs. One-way or two-way ANOVA with the appropriate post-hoc tests were performed, with p< 0.05 indicating significance.

## Results

### NOD MSCs conform with the ISCT classification criteria

**Fig 1:**
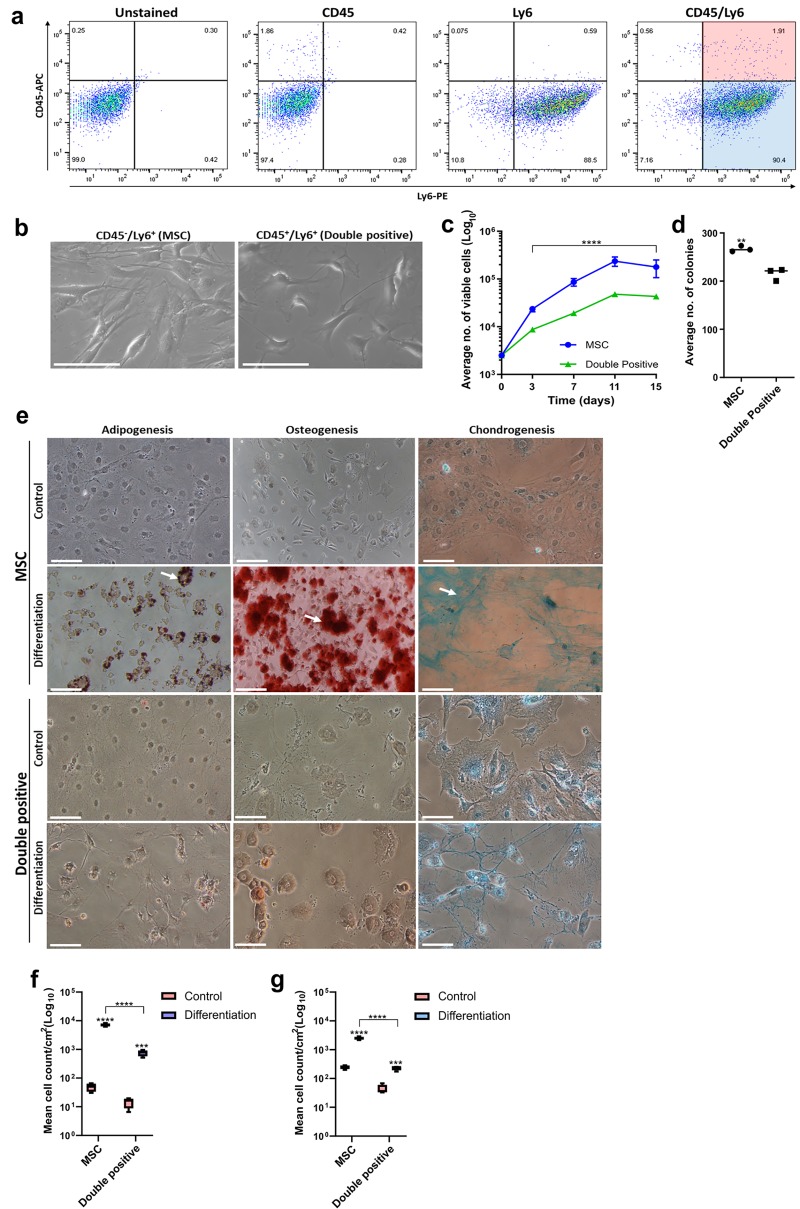
FACS enrichment and functional characterization of NOD MSCs. (a) FACS enrichment of NOD MSCs. Following culture for two passages, NOD bone marrow stromal cells were stained with CD45 mAb conjugated to fluorochrome APC (CD45-APC), Ly6 MAb conjugated to fluorochrome PE (Ly6-PE) and both mAbs (CD45-APC/Ly6-PE). Unstained cells were used as a negative control. Fluorescence dot plots of CD45-APC (y-axis) and Ly6-PE (x-axis) were used to identify the MSC (CD45^-^/Ly6^+^; blue) and double positive (CD45^+^/Ly6^+^; red) cell subpopulations ready for cell sorting using the BD *FACSAria II* flow cytometer. Data are representative of two individual FACS sorting experiments; (b) Plastic adherence, fibroblast-like morphology and self-renewal without differentiation into other cell types. MSCs maintained fibroblast-like morphology as assessed using light microscopy (Leica DM microscope; 10x magnification; scale bar = 100μM); (c) Improved cell proliferation with culture expansion. Data are presented as mean viable cells ± SDs (n = 3). A two-way ANOVA with Tukey’s post tests were performed, *p<0.05; (d) Improved fibroblastic colony formation following Methylene blue staining. Data are presented as mean number of colonies ± SEMs (n = 3). A one-way ANOVA and Tukey’s post tests were performed, * p<0.05; (e) Tri-lineage differentiation assays. For adipogenesis, mature and immature adipocytes are stained with Oil Red O, and indicated by white and yellow arrowheads respectively. For osteogenesis, osteocytes are stained with Alizarin Red, and indicated by white arrowheads. For chondrogenesis, filamentous glycosaminoglycans of chondrocytes are stained with Alcian blue, and are indicated by white arrowheads. Images were acquired on a Nikon Eclipse TS2 microscope at 20x magnification, 100μm scale; (f) Semi-quantitative analysis of adipogenic differentiation under defined conditions. Data are presented as mean cell count/cm^2^ ± SEM (n = 3). A two-way ANOVA and Tukey’s post tests were performed, * p<0.05; (f) Semi-quantitative analysis of osteogenic differentiation under defined conditions. Data are presented as mean cell count/cm^2^ SEM (n = 3). A two-way ANOVA and Tukey’s post tests were performed, * p<0.05.

MSCs identified by FACS correspond to the CD45^-^/Ly6^+^ cell population, which constituted ~80–90% of the parental stromal cell population (**Fig 1A**). These cells display plastic adherence and a fibroblast-like morphology (**Fig 1B**), unlike CD45^+^/Ly6^+^ (double positive) cells, which appear to possess an irregular cuboidal morphology. An inter-population analysis of cell proliferation showed that MSCs possess enhanced proliferation by comparison to double positive cells (**Fig 1C**). In addition, MSCs demonstrated enhanced clonogenicity by comparison to double positive cells (**Fig 1D**). Following preliminary culture of the two sorted cell populations, tri-lineage differentiation assays were performed to confirm the functional identity of the sorted CD45^-^/Ly6^+^ cells as MSCs. Oil Red O, Alizarin Red and Alcian Blue staining of adipogenesis, osteogenesis and chondrogenesis respectively demonstrated that sorted MSCs possessed tri-lineage differentiation potential (**Fig 1E**). An inter-population analysis of adipogenic (**Fig 1F**) and osteogenic (**Fig 1G**) differentiation showed that MSCs possess enhanced differentiation potential by comparison to double positive cells. As a result, CD45^-^/Ly6^+^ enriched cells correspond to MSCs as defined by the International Society for Cellular Therapy (ISCT) [4, 24].

**Fig 2:**
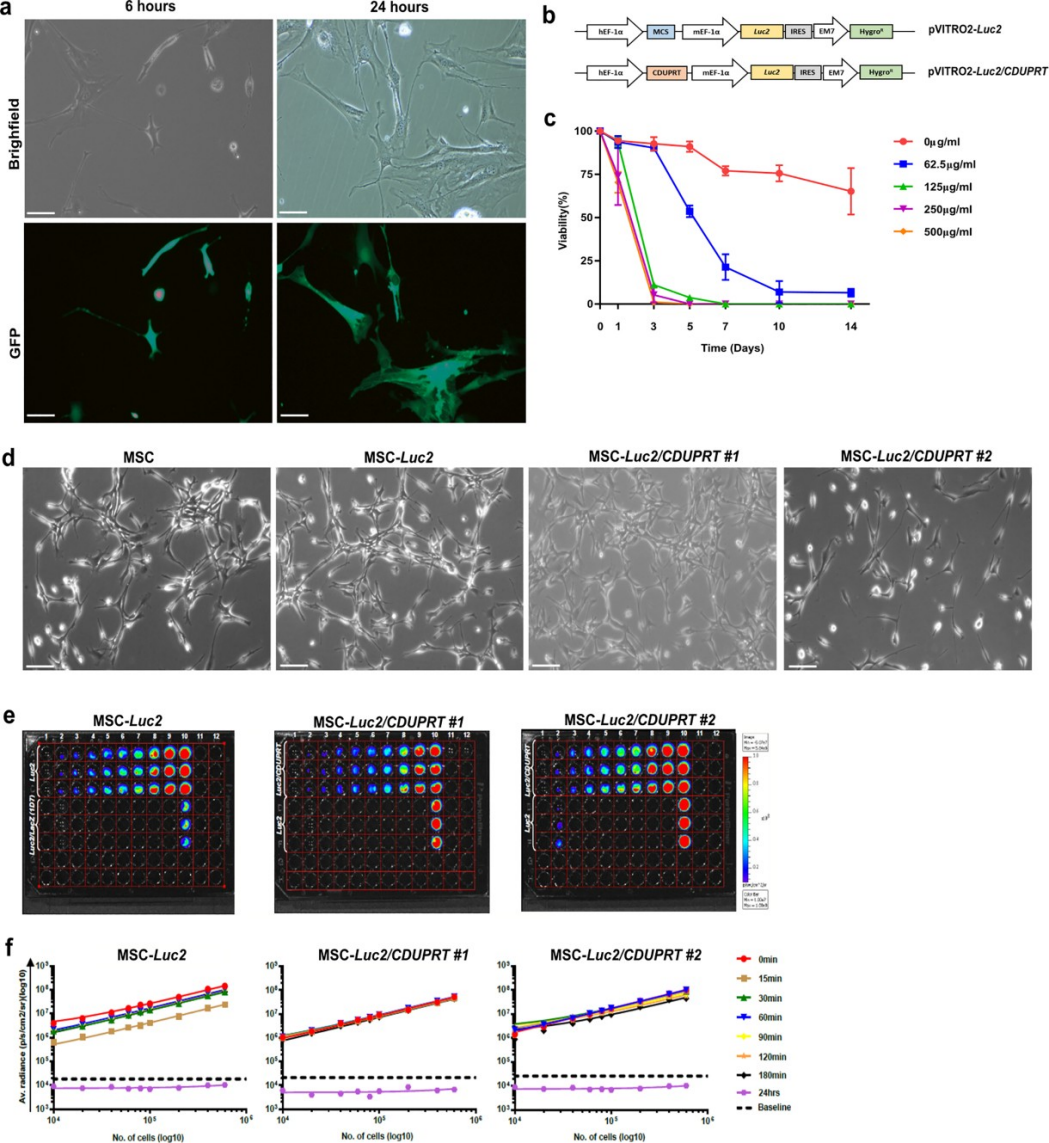
Morphological analysis of nucleofected MSCs. (a) Nucleofection with pmaxGFP. MSCs were nucleofected with pmaxGFP and imaged at 6 and 24 hours post nucleofection. Brightfield and fluorescent images were obtained using a Nikon Eclipse TS2 microscope at 20x magnification, 100μm scale. (b) Schematic representation of the pVITRO2-Luc2 and pVITRO2-Luc2/CDUPRT mammalian expression plasmids. (c) Antibiotic sensitivity assay. Native MSCs were grown in the presence of a 2-fold serial dilution of Hygromycin B (0–500μg/ml) for 14 days. (d) Nucleofection with bioluminescent plasmids. MSCs were nucleofected with pVITRO2-*Luc2* and pVITRO2-*Luc2/CDUPRT*, and selected for with Hygromycin B for two weeks. Images were acquired on a Leica DM Microscope at 10x magnification, 100μm scale. (e) BLI in MSC-*Luc2*, MSC-*Luc2/CDUPRT #1* and MSC-*Luc2/CDUPRT #2* over a linear cell concentration range. The cells were incubated with 1:1 D-luciferin (300μg/ml) and imaged on the IVIS Lumina II according to the *in vitro* BLI acquisition settings. The images are represented at t = 30min. Lane 1: D-PBS, Lane 2–10: Nucleofected MSC vs control MSC. (f) Analysis of luciferase activity in MSC-*Luc2*, MSC-*Luc2/CDUPRT #1* and MSC-*Luc2/CDUPRT #2*. Luminescence was captured at multiple time-points following incubation with D-luciferin was analyzed using GraphPad Prism 7. Data are represented as the mean average radiance ± SDs of triplicates.

MSCs nucleofected with pmax-GFP and analyzed by fluorescence microscopy showed that ~50% of MSCs were GFP^+^ at 6 hours post-nucleofection, which increased to ~70–75% GFP^+^ at 24 hours post-nucleofection (**Fig 2A**). Variations in GFP fluorescence intensity were also observed amongst GFP^+^ MSCs. To generate bioluminescent/reporter MSCs (MSC-*Luc2*) and suicide/therapeutic MSCs (MSC-*Luc2*/*CDUPRT*), early passage MSCs were nucleofected with pVITRO2-*Luc2* and pVITRO2-*Luc2*/*CDUPRT* (**Fig 2B**) respectively. The nucleofection efficiency could not be quantified due to the absence of a co-expressed fluorescent reporter for downstream analysis. An antibiotic sensitivity assay for Hygromycin B determined that the concentration of antibiotic required to kill >90% of native MSCs within 7–10 days was 200μg/ml (**Fig 2C and S1 Table**). Following nucleofection with pVITRO2-*Luc2* and pVITRO2-*Luc2*/*CDUPRT*, Hygromycin B selection yielded one and two stable clones respectively. Morphological analysis showed that by comparison to parental MSCs, nucleofected MSCs retained a fibroblast-like morphology despite a reduced cytoplasmic volume (**Fig 2D**).

### Luciferase is a stable reporter of MSC viability *in vitro*

*In vitro* characterization of luciferase activity was assessed in bioluminescent MSCs, and suicide MSCs clone 1 and clone 2 via BLI at multiple time-points following incubation with D-luciferin (**Fig 2E**). An increase in luminescent signal is observed in all *Luc2*-expressing MSC clones with increasing cell density. The decrease in luminescent signal emitted from the positive control MSC-*Luc2/LacZ ID7* at equivalent cell numbers is likely attributable to clonal differences in *Luc2* expression. Analysis of luminescent signal represented as average radiance (photons/sec/cm^2^/sr), demonstrated a linear correlation between cell concentration and units of luminescence in each of the *Luc2*-expressing MSC cell lines. All *Luc2*-expressing MSC clones demonstrated R^2^ values of >0.98 at each time point between t = 0 and t = 3 hours. The linear correlation between cell concentration and luminescence confirmed the selection of clonal populations of *Luc2*-expressing MSC (**Fig 2F and S2 Table**). In addition, the overlapping luminescence curves at multiple time-points demonstrated stability in the luminescent signal up to 3-hours following incubation with D-luciferin. No significant difference was observed in the luminescent signal between bioluminescent MSCs and the two suicide MSC clones. At 24 hours following addition of D-luciferin, luminescence from bioluminescent MSCs and the two suicide MSC clones fell below the baseline (background) luminescence. Due to the improved stability of luminescence determined by tightly overlapping luminescence curves of suicide MSC clone 1 in comparison to clone 2, clone 1 was utilized in the cytotoxicity assays.

### *CDUPRT*-expressing MSCs demonstrate enhanced cytotoxicity *in vitro*

**Fig 3:**
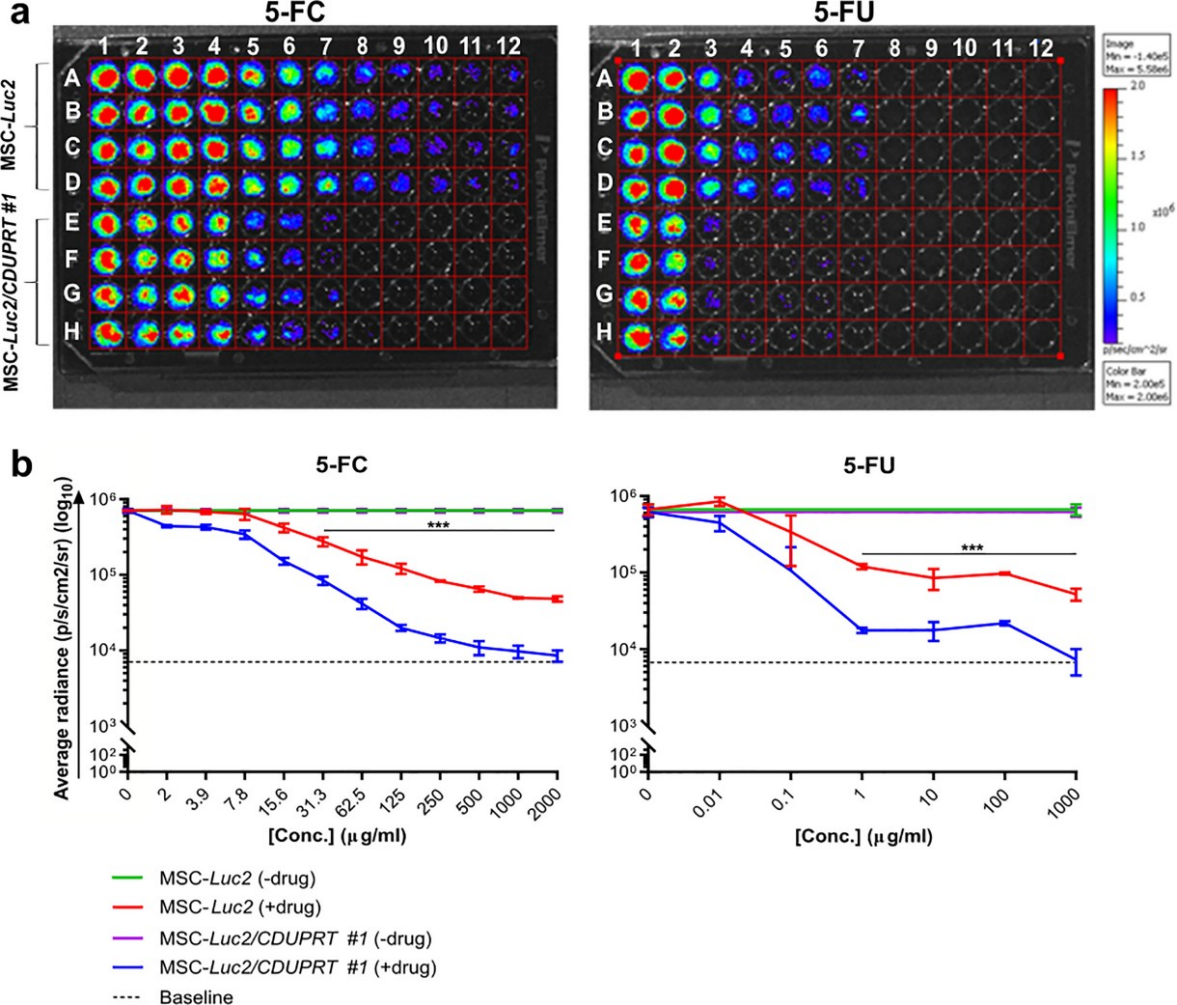
*In vitro* luciferase-based cytotoxicity assay. (A) *In vitro* BLI of *CDUPRT* activity. BLI was assessed following the addition of 5-FC and 5-FU to MSC-*Luc2* (A-D) and MSC-*Luc2/CDUPRT #1* (E-H). For 5-FC BLI data: Lanes 1–12 contains 2-fold serial dilutions of 5-FC from 0–2000μg/ml. For 5-FU BLI data: Lanes 1–7 contains 10-fold serial dilutions of 5-FU from 0–1000μg/ml. (B) Analysis of BLI data. Data are represented as the mean average radiance ± SEMs (n = 3). Baseline luminescence is indicated by a dotted-line. A two-way ANOVA and Sidak’s post-hoc were performed, p<0.05.

Functional *CDUPRT* cytotoxicity was assessed in suicide/therapeutic MSCs compared with bioluminescent/reporter MSC following the addition of 5-FC (0–2000μg/ml) via BLI (**Fig 3A**). General 5-FU cytotoxicity was assessed following the addition of 5-FU (0–1000μg/ml) via BLI. Bioluminescence correlates to cell viability due to the functional requirement for ATP for luciferase activity [17], and therefore a decrease in bioluminescence correlates with a decrease in cell viability. Luminescence analysis showed a significant decrease in bioluminescence from suicide/therapeutic MSCs in comparison with reporter MSCs following the addition of 5-FC ranging from 31.25–2000μg/ml (p<0.005), confirming functional *CDUPRT* activity in suicide/therapeutic MSCs (**Fig 3B**). The concentrations at which there was a >90% reduction in bioluminescent signal following addition of 5-FC for bioluminescent/reporter MSCs and suicide/therapeutic MSCs in comparison to their untreated controls were 500μg/ml and 31.25μg/ml respectively, highlighting the enhanced cytotoxicity of suicide/therapeutic MSCs. A similar trend in enhanced cytotoxicity of suicide/therapeutic MSC was observed following the addition of 5-FU, where there was a significant decrease in bioluminescence from suicide/therapeutic MSCs in comparison to reporter MSCs, ranging from 1–1000μg/ml (p<0.005). The concentrations at which there was a >90% reduction in the bioluminescent signal following the addition of 5-FU for bioluminescent/reporter MSCs and suicide/therapeutic MSCs in comparison to their untreated controls were 1000μg/ml and 1μg/ml respectively. Thus, at equivalent concentrations, 5-FU demonstrates higher toxicity than 5-FC in suicide/therapeutic MSCs. Furthermore, it was demonstrated that >15.6μg/ml 5-FC was toxic to reporter MSCs with a significant decrease (p<0.01) in bioluminescence observed when compared to untreated control MSC.

## Discussion

MSC isolation from a variety of tissue sources has been attempted using a number of methodologies including antibody-based cell sorting [25], low and high-density culture [26], positive and negative selection [27], frequent media changes [28] and enzymatic digestion [29]. In this study, we report the enrichment of NOD MSCs using CD45^-^/Ly6^+^ FACS sorting for subsequent *ex vivo* expansion and gene modification. Using this FACS sorting technique, we isolated a sub-population of NOD MSCs that displayed the characteristic fibroblast-like phenotype and tri-lineage differentiation potential of MSC as defined by the ISCT MSC [4, 24]. In addition, the enriched MSCs constituted ~80–90% of the initial adherent bone marrow stromal cell population, a significant improvement by comparison to similar studies [30–33].

In this study, nucleofection was utilized as a non-viral method of MSC gene modification due to its reported success in modifying MSCs without affecting proliferation, phenotype or differentiation potential [34, 35]. Nucleofection of MSCs with the pmaxGFP plasmid showed a transfection efficiency of ~70% at 24 hours post-nucleofection which is similar to that reported in the literature [34, 35]. In fact, nucleofection out performs other transfection methods such as calcium phosphate precipitation, cationic polymer and standard electroporation with respect to gene-modification of MSCs [34, 36]. For the purpose of short-term/transient ectopic gene expression in difficult to transfect cells such as adult stem cells, nucleofection is a suitable alternative to traditional physical, non-viral methods of gene-modification. However, despite this success, variations in the transduction efficiency of MSCs have been observed across species [37]. In addition, stable transfection as a consequence of successful genomic integration is limited by the poor rate of integration (600 per million cells (0.06%)) with a ~5kb plasmid. With increasing plasmid size, integration events decrease further [38]. As a result, due to higher transduction efficiency and genomic integration events, viral-mediated transduction remains the mainstay in generating gene-modified cells that stably express transgenes of interest [39–41].

The *in vitro* characterization of luciferase expression from stably selected bioluminescent MSCs and suicide MSCs was equivalent. Due to the ATP requirement of luciferase for light emission, luciferase expression was utilized as a reporter for cell survival during the *in vitro* characterization of *CDUPRT* function. The ability of *CDUPRT* to convert a non-toxic concentration of 5-FC to the toxic metabolite 5-FU was assessed by exposing bioluminescent/reporter MSCs and suicide/therapeutic MSCs to various concentrations of 5-FC *in vitro*. Suicide MSCs demonstrated a significant decrease in cell survival when compared with bioluminescent MSCs following the addition of >31.3ug/mL 5-FC, confirming the *in vitro* functional activity of *CDUPRT*. In fact, at equivalent doses, 5-FU demonstrated significantly higher toxicity than 5-FC in cells expressing *CDUPRT*. This is due to the combined inhibition of DNA and RNA synthesis in MSC-*Luc2*/*CDUPRT* as opposed to DNA inhibition alone in bioluminescent MSCs following addition of 5-FU. Research conducted in rat prostate adenocarcinoma cells that were transduced to express the *CDUPRT* gene showed similar results [42]. In addition, the known metabolic pathway involved in the conversion of 5-FC to 5-FU and its toxic metabolites supports the results of this study [43, 44]. Due to the intracellular requirement of *CD* to convert 5-FC to 5-FU prior to the generation of 5-FU toxic metabolites, additional steps (both mechanical and enzymatic) are required for 5-FC killing of *CDUPRT*-expressing cells in comparison to 5-FU. As a result, 5-FC toxicity is likely affected by its rate of cellular uptake, *CD* conversion and degradation; resulting in the observed differences in toxicity of 5-FC and 5-FU in *CDUPRT*-expressing cells. 5-FU on the other hand is membrane permeable and does not require the rate limiting step of *CD* conversion to a toxic metabolite.

**Fig 4:**
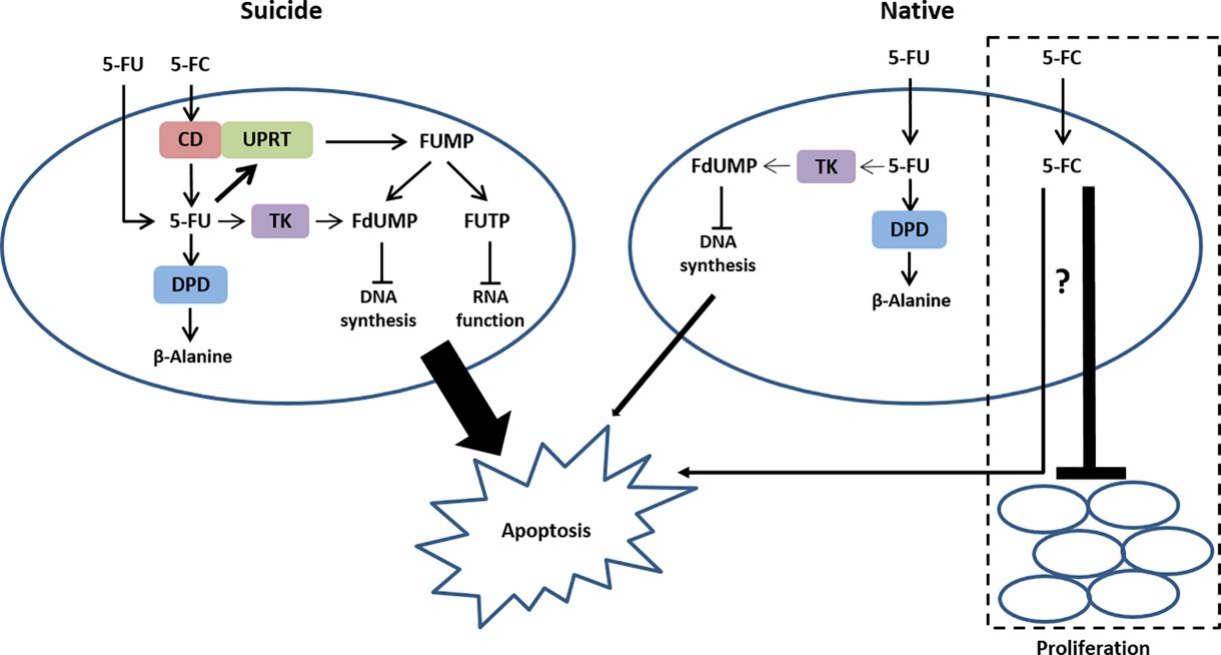
Mechanism of action of 5-FC and 5-FU in MSCs. In cells expressing *CDUPRT*, 5-FC is transported into the cell where it is converted into 5-FU by the *CD* component of *CDUPRT*. *UPRT* subsequently converts 5-FU into FUMP which is further processed into FdUMP and FUTP which inhibit DNA synthesis and RNA function respectively, leading to apoptosis. Endogenous DPD degrades 5-FU into non-toxic β-Alanine, which ultimately becomes the rate-limiting step in the conversion of 5-FU to its toxic metabolites. In cells that do not express *CDUPRT*, 5-FU toxicity is mediated by direct conversion to FdUMP via TK, leading to inhibition of DNA synthesis and apoptosis. In the absence of *CDUPRT*, 5-FC cannot be converted to 5-FU and may result in the inhibition of cell proliferation or cytotoxicity through an unknown mechanism. Thickness of arrows is reflective of the predominating pathway or effect. Abbreviation: *CD*UPRT; cytosine deaminase::uracil phosphoribosyltransferase, *CD*; cytosine deaminase, FUMP; 5-fluorouridine monophosphate, FdUMP; 5-fluorodeoxyuridine monophosphate, FUTP; 5-fluorouridine triphosphate, DPD; dihydropyrimidine dehydrogenase.

However, of particular interest was the effect of 5-FC on parental MSCs that do not express the *CD* or *CDUPRT* genes, which are necessary for 5-FC conversion to 5-FU [45], and as such would not be susceptible to 5-FC mediated toxicity. Most data on the effect of 5-FC on mammalian cells that are engineered to express *CD* or *CDUPRT* are expressed as a function of cytotoxicity, which as expected increases in comparison to cells that do not express *CD* or *CDUPRT* [42, 43]. Following the addition of 5-FC to bioluminescent MSCs that do not express *CDUPRT*, the observed decrease in bioluminescence in comparison to untreated bioluminescent MSCs suggested that 5-FC may be involved in the inhibition of MSC proliferation, or demonstrate cytotoxic effects at high doses, leading to a redefinition of the metabolic pathway of 5-FC and 5-FU in mammalian cells (**Fig 4**). In a similar study by Harrell *et al* [46] using primary vascular smooth muscle cells (VSMCs), treatment of parental VSMCs with 5-FC at a single concentration of 1mmol/L (equivalent to 129μg/ml) did not result in a significant difference in cell numbers in comparison to untreated parental VSMCs, suggesting the absence of a cytotoxic effect of 5-FC on VSMCs. This is surprising considering that we observed a >50% reduction in the viability of control MSC in comparison to untreated MSCs. In addition, the lack of a dose-response curve in the Harrell study failed to determine whether a cytotoxic effect would be observed at higher concentrations of 5-FC. Thus, the results reported in our study may be explained by improved experimental design and cell-type dependent sensitivity to 5-FC. Ultimately, this study demonstrates that luciferase is a suitable reporter of cell viability, and that MSCs have the potential to be utilized as vehicles of suicide gene therapy.

## Conclusions

This study showed luciferase reporter assays represent a novel method of quantitatively assessing *CDUPRT*-mediated cytotoxicity in genetically modified MSC. In addition, their translation to the *in vivo* setting facilitates pre-clinical cytotoxicity studies related to a variety of chronic diseases including cancer, neurodegenerative disorders, and diabetes. The results of this study demonstrated that *CDUPRT*-expressing MSCs become cytotoxic following administration of the non-toxic prodrug 5-FC. Thus, due to their tumor-homing properties [47, 48], a systemic infusion of *CDUPRT*-expressing MSCs as vehicles of suicide gene therapy may be useful for the treatment of a variety of cancers. In addition, *CDUPRT*-mediated cytotoxicity has the potential to be utilized as a clinical fail-safe switch to improve the safety of cell replacement therapies.

## Supporting information

S1 Table Statistical analysis of antibiotic sensitivity. (PDF)

S2 Table Statistical analysis of luciferase stability. (PDF)
