# Supplementary material for: Luciferase-based reporting of suicide gene activity in murine mesenchymal stem cells
Source: PLoS One. 2019 Jul 18;14(7):e0220013. doi: 10.1371/journal.pone.0220013 (PMC6638968; doi:10.1371/journal.pone.0220013)
Supplement: S1 Table — (PDF) [file pone.0220013.s001.pdf]

S1 Table. Statistical analysis of antibiotic sensitivity

Day 0					
	**0**	**62.5**	**125**	**250**	**500**
**0**		ns	ns	ns	ns
**62.5**	ns		ns	ns	ns
**125**	ns	ns		ns	ns
**250**	ns	ns	ns		ns
**500**	ns	ns	ns	ns	

Day 1					
	**0**	**62.5**	**125**	**250**	**500**
**0**		ns	ns	ns	ns
**62.5**	ns		ns	ns	*
**125**	ns	ns		ns	*
**250**	ns	ns	ns		ns
**500**	ns	*	*	ns	

Day 3					
	**0**	**62.5**	**125**	**250**	**500**
**0**		ns	***	***	***
**62.5**	ns		****	***	****
**125**	***	****		ns	***
**250**	***	***	ns		ns
**500**	***	****	***	ns	

Day 5					
	**0**	**62.5**	**125**	**250**	**500**
**0**		****	****	****	****
**62.5**	****		****	****	****
**125**	****	****		****	****
**250**	****	****	****		ns
**500**	****	****	****	ns	

Day 7					
	**0**	**62.5**	**125**	**250**	**500**
**0**		****	****	****	****
**62.5**	****		****	****	****
**125**	****	****		ns	ns
**250**	****	****	ns		ns
**500**	****	****	ns	ns	

Day 10					
	**0**	**62.5**	**125**	**250**	**500**
**0**		****	****	****	****
**62.5**	****		****	****	****
**125**	****	****		ns	ns
**250**	****	****	ns		ns
**500**	****	****	ns	ns	

Day 14					
	**0**	**62.5**	**125**	**250**	**500**
**0**		****	****	****	****
**62.5**	****		****	****	****
**125**	****	****		ns	ns
**250**	****	****	ns		ns
**500**	****	****	ns	ns	

Values are represented as means ± SDs or SEMs. One-way ANOVA with the appropriate post-hoc tests were performed, with * $p < 0.05$ indicating significance.
