# Supplementary material for: Luciferase-based reporting of suicide gene activity in murine mesenchymal stem cells
Source: PLoS One. 2019 Jul 18;14(7):e0220013. doi: 10.1371/journal.pone.0220013 (PMC6638968; doi:10.1371/journal.pone.0220013)
Supplement: S2 Table — (PDF) [file pone.0220013.s002.pdf]

S2 Table. Statistical analysis of luciferase stability

MSC-*Luc2*								
	**0min**	**15min**	**30min**	**60min**	**90min**	**120min**	**180min**	**24hrs**
**R square**	0.9961	0.9984	0.9991	0.9997	0.9991	0.9981	0.9966	0.5706
**P value**	<0.0001	<0.0001	<0.0001	<0.0001	<0.0001	<0.0001	<0.0001	0.0186
**Deviation from zero?**	Significant	Significant	Significant	Significant	Significant	Significant	Significant	Significant
**Equation**	Y = 243.1*X + 2227984	Y = 40.31*X + 115631	Y = 133.4*X + 425745	Y = 167.1*X + 348163	Y = 175.9*X - 2273	Y = 159.8*X + 332401	Y = 138.3*X + 257333	Y = 0.004618*X + 7445

MSC-*Luc2/CDUPRT #1*								
	**0min**	**15min**	**30min**	**60min**	**90min**	**120min**	**180min**	**24hrs**
**R square**	0.9948	0.9988	0.9996	0.9999	0.9999	0.9989	0.9998	0.2231
**P value**	<0.0001	<0.0001	<0.0001	<0.0001	<0.0001	<0.0001	<0.0001	0.1992
**Deviation from zero?**	Significant	Significant	Significant	Significant	Significant	Significant	Significant	Not Significant
**Equation**	Y = 82.69*X + 120942	Y = 74.31*X + 433921	Y = 82.37*X + 411866	Y = 86.93*X + 346359	Y = 80.28*X + 363460	Y = 77.54*X + 409999	Y = 71.27*X + 42897	Y = 0.003578*X + 4973

MSC-*Luc2/CDUPRT #2*								
	**0min**	**15min**	**30min**	**60min**	**90min**	**120min**	**180min**	**24hrs**
**R square**	0.999	0.9988	0.991	0.9897	0.9816	0.9611	0.9717	0.8606
**P value**	<0.0001	<0.0001	<0.0001	<0.0001	<0.0001	<0.0001	<0.0001	0.0003
**Deviation from zero?**	Significant	Significant	Significant	Significant	Significant	Significant	Significant	Significant
**Equation**	Y = 169.6*X + 59007	Y = 151.4*X + 1059817	Y = 149.0*X + 2348173	Y = 133.9*X + 2040637	Y = 120.3*X + 2400597	Y = 101.2*X + 2541196	Y = 79.05*X + 1538026	Y = 0.04554*X + 2558

Values are represented as means ± SDs or SEMs. One-way ANOVA with the appropriate post-hoc tests were performed, with * $p < 0.05$ indicating significance.
